# Supplementary material for: Matching sensor ontologies through siamese neural networks without using reference alignment
Source: PeerJ Comput Sci. 2021 Jun 18;7:e602. doi: 10.7717/peerj-cs.602 (PMC8237319; doi:10.7717/peerj-cs.602)
Supplement: Supplemental Information 1 [file peerj-cs-07-602-s001.zip › 254/refalign.html]

# (level 0) Alignment

## Source: http://oaei.ontologymatching.org/2011/benchmarks/101/onto.rdf

## Target: http://oaei.ontologymatching.org/2011/benchmarks/254/onto.rdf

## Correspondences

PersonList = dsqdbz
:   1.0

Unpublished = zeadza
:   1.0

Address = qzddj
:   1.0

Chapter = dzqndbzq
:   1.0

InBook = vccfsq
:   1.0

Date = dzshyuua
:   1.0

PageRange = dnqsb
:   1.0

Booklet = zdzaudbd
:   1.0

LectureNotes = scds
:   1.0

MastersThesis = xsqlknk
:   1.0

TechReport = sdcsqhyz
:   1.0

Misc = wxsdxqsdh
:   1.0

Collection = sqxsqkd
:   1.0

Academic = zdazsx
:   1.0

MotionPicture = dscdscg
:   1.0

Article = hazdn
:   1.0

Informal = qsdsnbsqd
:   1.0

Book = zajdhqs
:   1.0

School = zadazxn
:   1.0

PhdThesis = zqdzqzai
:   1.0

Proceedings = zdqssqdb
:   1.0

Reference = sqdsq
:   1.0

InCollection = dcsqdcsqd
:   1.0

Report = dqzdxdcsqj
:   1.0

Conference = zqedzbx
:   1.0

Part = sqdsopq
:   1.0

Publisher = zauio
:   1.0

Manual = dcsqdsq
:   1.0

InProceedings = deqdxcsqcsq
:   1.0

Journal = qsdsquj
:   1.0

Monograph = dzajndsq
:   1.0

Institution = izxnquo
:   1.0

Deliverable = azzauhuie
:   1.0
